# Supplementary material for: Microbial mineralization of cellulose in frozen soils
Source: Nat Commun. 2017 Oct 27;8:1154. doi: 10.1038/s41467-017-01230-y (PMC5658388; doi:10.1038/s41467-017-01230-y)
Supplement: Supplementary file 1 — Supplementary Information [file 41467_2017_1230_MOESM1_ESM.pdf]

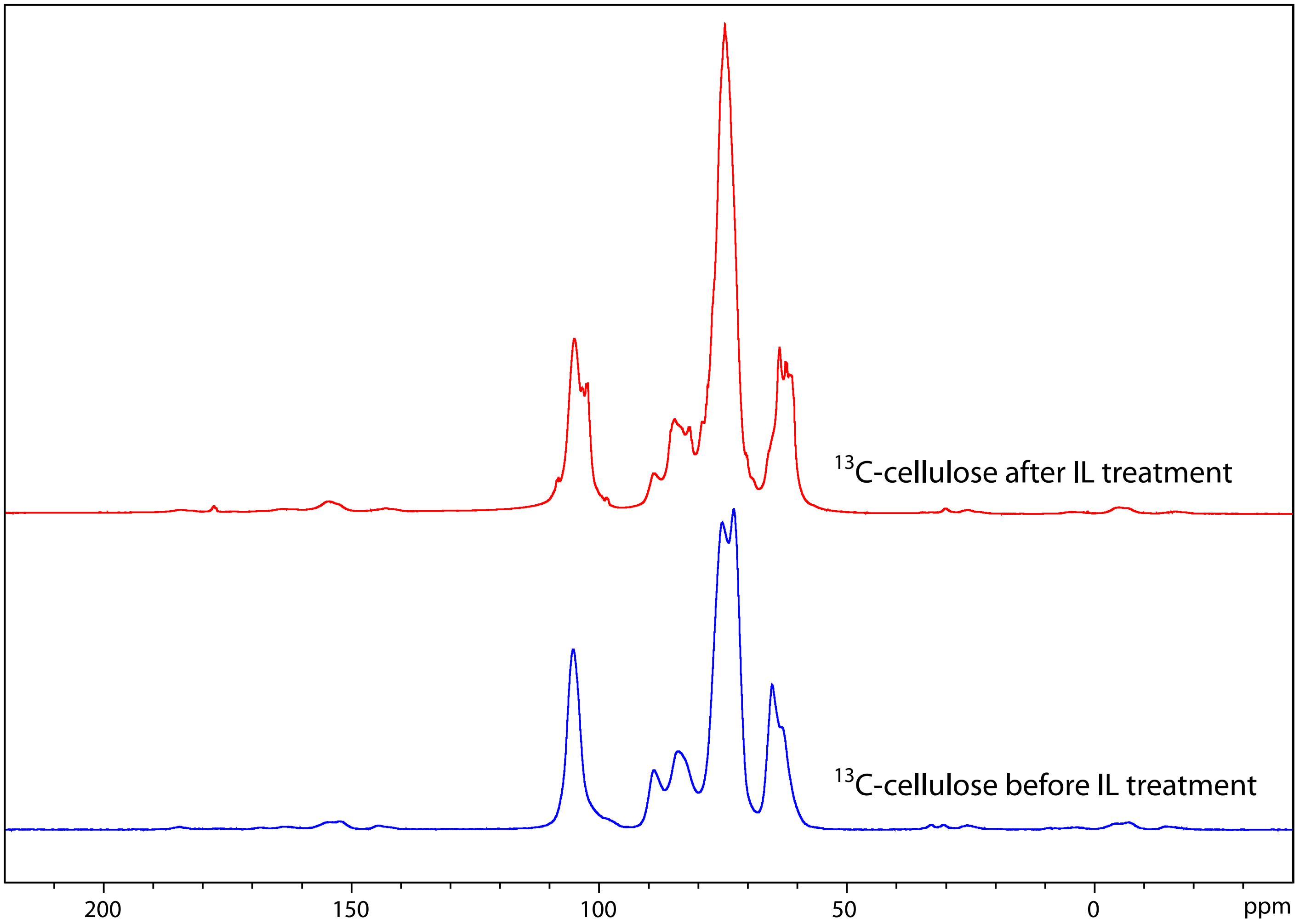

Supplementary figure 1. **CP-MAS NMR spectra of the ^{13}C-labelled cellulose before and after ionic liquid treatment.** The shift in C4 carbon of the cellulose at 90 to 95 ppm indicates the transformation of the crystalline cellulose into amorphous cellulose.

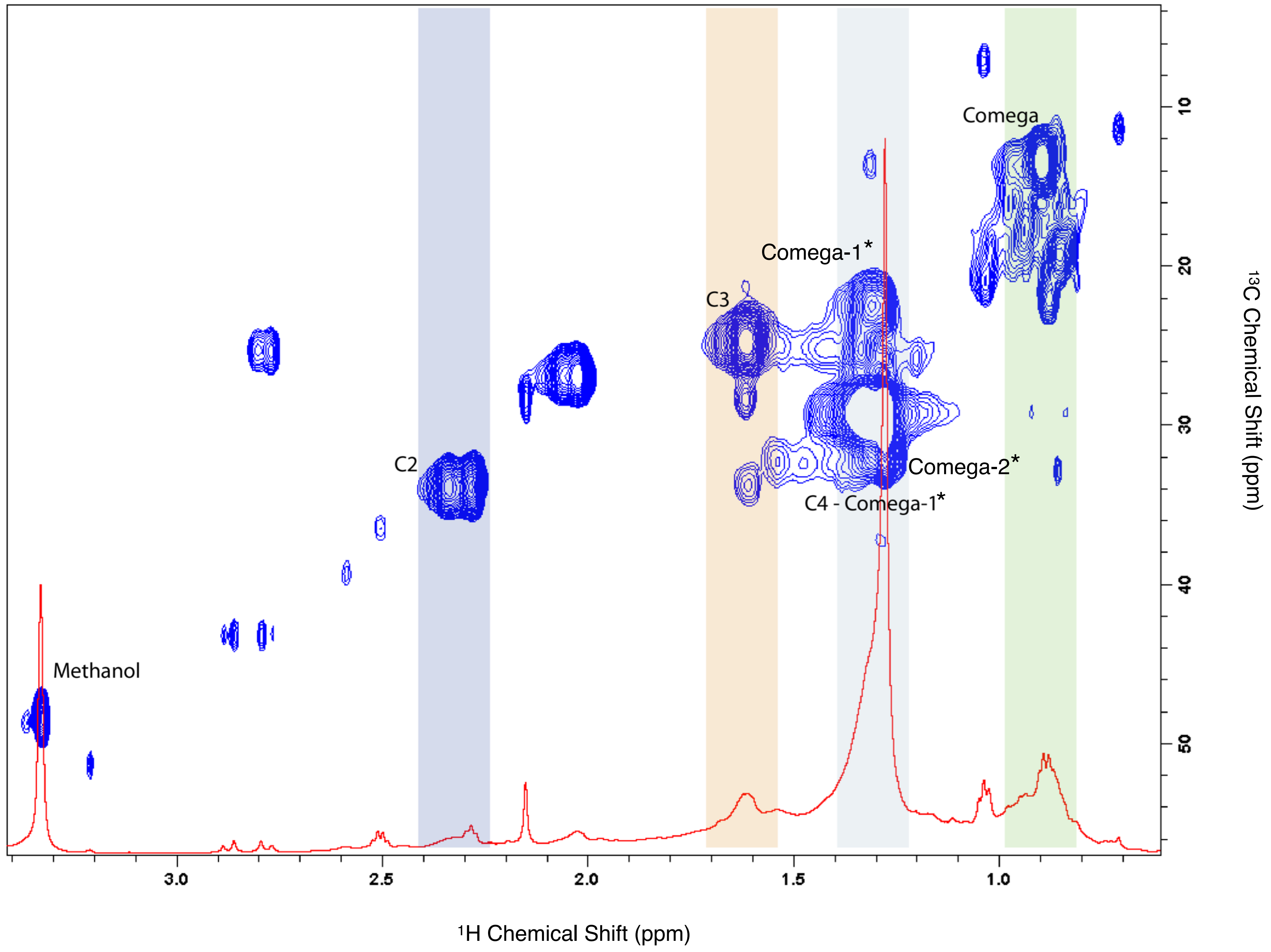

Supplementary Figure 2. 2D HSQC spectrum of a representative soil lipid extract in chloroform/methanol overlaid with the corresponding 1D ^{1}H spectrum. The colored stripes indicate the ^{1}H projections interpreted as lipid chain signals C2, C3, C4 - CΩ-1 and CΩ, respectively. The residual ^{1}H signal due to deuterated methanol solvent is also indicated. This particular sample also contains ethylmethylketone (99.5 atom %, Kebo lab, Spånga, Sweden) as an internal standard, with e.g. the methyl peak at (2.15 ppm, 28.3 ppm). No overlap is visible for the C2 signal and the C4 - CΩ-1 strip is also totally dominated by the lipid signal (see asterisk Supplementary Figure 2). For C3 and CΩ, the signal overlap corresponds to ca. 20%.
